# Effects of early enteral micro-feeding on neonatal serum Vitamin D levels

**DOI:** 10.12669/pjms.316.8886

**Published:** 2015

**Authors:** Liang Hu, Xiangdang Yin, Haifeng Chu, Guangli Zheng

**Affiliations:** 1 Liang Hu, Child’s Hospital of Changchun, Changchun 130051, China; 2 Xiangdang Yin, Jilin Cancer Hospital, Changchun 130001, China; 3 Haifeng Chu, China-Japan Union Hospital, Jilin University, Changchun 130033, China; 4 Guangli Zheng, Child’s Hospital of Changchun, Changchun 130051, China

**Keywords:** Enteral feeding, Glutamine, Neonate, Serum vitamin D, Gastrointestinal hormone

## Abstract

**Objective:**

To evaluate the effects of early enteral micro-feeding on neonatal serum vitamin D levels, and to analyze the application value of glutamine.

**Methods:**

One hundred ninty neonates enrolled in intensive care unit were randomly divided into a treatment group and a control group (n=95) that were both given enteral and parenteral nutrition support. Meanwhile, the treatment group was fed formula milk containing 0.3 g/(kg·d) glutamine as enteral nutrition support for 14 days.

**Results:**

The weight of the treatment group increased significantly faster than that of the control group did (P<0.05). The treatment group had significantly higher milk amount and calorie intake than those of the control group (P<0.05), and neonates in the treatment group who reached calorie intake of 50/80/100 kcal/kg/d were significantly younger (P<0.05). Meanwhile, the treatment group was significantly less prone to feeding intolerance than the control group (P<0.05). After 14 days of feeding, the serum motilin, gastrin and vitamin D levels of both groups all increased, with significant intra-group and inter-group differences. Such levels of the treatment group significantly exceeded those of the control group (P<0.05).

**Conclusion:**

Supplementing early enteral micro-feeding with glutamine promoted the absorption of neonatal routine nutrients and vitamin D, obviously regulated gastrointestinal hormones, and elevated weight as a result.

## INTRODUCTION

The survival rates of neonates, especially those of premature infants, continuously increase with the development of nursing technology. However, many neonates suffer from feeding intolerance that is mainly manifested as vomiting, abdominal distension and increased gastric residue, thus affecting gastrointestinal caloric intake and leading to protein deficiency, weight stasis or even decrease, and low quality of life. Therefore, it is of great significance to facilitate the development and maturation of the gastrointestinal tract in order to enhance feeding tolerance.^1^,^2^

Since premature infants are prone to many severe diseases such as asphyxia, gastrointestinal tract abnormalities, necrotizing enterocolitis and respiratory distress syndrome, their gastrointestinal tracts fail to effectively absorb nutrients, grow and develop slowly, and even have mucosal atrophy.^3^ Vitamin D2 (ergocalciferol) and vitamin D3 (cholecalciferol), collectively known as vitamin D, is an important nutrient that essentially controls regulation of calcium and phosphorus metabolisms as well as regulates immunity, cell growth and differentiation.^4^,^5^ It is well known that early micro-feeding can stimulate the development and maturation of the gastrointestinal tract, promote release of gastrointestinal hormones and augment gastrointestinal motility, thereby increasing the feeding tolerance and development of neonates.^6^,^7^

Basic and animal experiments have verified that glutamine can effectively improve the nutritional status of children, enhance cellular immunity and intestinal mucosal barrier function,^8^ protect the intestinal mucosa integral, and maintain normal intestinal permeability. Providing nitrogen and energy simultaneously, glutamine is the precursor for pyrimidine synthesis and also accelerates cell proliferation and differentiation.^9^,^10^ In this study, we evaluated the effects of early enteral micro-feeding on neonatal serum vitamin D levels, and analyzed the application value of glutamine.

## METHODS

### Subjects

A total of 190 neonates enrolled in the intensive care unit of our hospital from September 2009 to February 2014 were selected. This study has been approved by the ethics committee of Child’s Hospital of Changchun.

### Inclusion criteria

Neonates who were enrolled within 72 hour after being born; single births; requirement of external nutrition support; without contraindications for gastrointestinal nutrition support; with consent from guardians.

### Exclusion criteria

With diagnosed or suspected congenital digestive, metabolic or chromosomal diseases; with abnormal liver and kidney functions; with congenital or acquired immune deficiency syndrome. They were then randomly divided into a treatment group and a control group (n=95), and their gender ratio, gestational age, birth weight, mode of delivery and 1 min, 5 minutes Apgar scores (P>0.05) (Table-I).

**Table-I:** Baseline clinical data.

Index	Treatment group (n=95)	Control group (n=95)	t or χ^2^	P
Gender (male/female)	51/44	50/45	0.078	>0.05
Gestational age (W)	37.92±3.13	37.88±3.98	0.127	>0.05
Birth weight (kg)	2.31±0.45	2.29±0.43	0.082	>0.05
Mode of delivery (vaginal birth/C-section)	34/61	32/63	0.210	>0.05
1 min Apgar score (point)	8.09±1.78	8.11±1.73	0.054	>0.05
5 min Apgar score (point)	9.03±1.14	9.05±1.34	0.068	>0.05

### Intervention methods

All neonates were nutritionally supported according to the Guideline for Clinical Practice of Nutrition Support in Chinese Neonates. During fasting, total parenteral nutrition was employed, which was gradually changed to partial parenteral and total enteral nutrition. Enteral micro-feeding was conducted by nurses. They were all fed formula milk specific to infants with low birth weights. Besides, 0.3g (kg·d) glutamine (Chonqging Yaoyou Pharmaceutical Co., Ltd.) was added in the milk of the treatment group to replace corresponding amino acids in conventional enteral nutrition prescription. Starting from 10 ml/kg/d, milk amount was gradually increased (no exceeding 20 ml/kg/d) based on gastric milk residue, vomiting, abdominal distention and bowel movement. Parenteral nutrition, which comprised fat emulsion, 6% pediatric amino acid solutions, glucose injection, electrolytes, vitamins and trace elements, was 24 hour infused at a constant rate by using the all-in-one mode through a micropump infusion set. Both groups were treated and observed for 14 consecutive days.

### Observation indices: Indices for neonatal development

The growth rates of head circumference, height and weight were observed and calculated.

### Feeding outcomes

The milk amount, calorie intake and ages in days on which 50, 80 and 100 kcal/kg/d calories were reached were observed and recorded. In the meantime, feeding intolerance symptoms, such as vomiting, abdominal distention and gastric retention, were investigated.

### Measurement of serum motilin (MOT) and gastrin (GAS) levels

Venous blood (2 ml) was drawn in the early morning on the 1st and 14th days of feeding, and centrifuged for 20 min, from which serum was collected into EP tubes containing anticoagulants, stored in -20°C refrigerator and detected by enzyme-linked immunosorbent assay (ELISA).

### Measurement of serum vitamin D level

Serum was separated with the method mentioned above, vitamin D level of which was measured by double antibody sandwich ELISA.

### Statistical analysis

All data were analyzed by SPSS13.0. The measurement data were expressed as mean ± standard deviation (x ± s), and inter-group comparisons were performed with independent samples t-test and analysis of variance. The numerical data between groups were compared by Chi-square test. P<0.05 was considered statistically significant.

## RESULTS

### Neonatal developmental statuses

The weight of the treatment group increased significantly faster than that of the control group did (P<0.05), but their head circumferences and heights grew at similar rates (Table-II).

**Table-II:** Neonatal developmental statuses.

Group	Case No. (n)	Growth rate of head circumference (cm/w)	Growth rate of height (cm/w)	Growth rate of weight (g/kg/d)
Treatment group	95	0.46±0.11	0.77±0.15	14.34±1.33
Control group	95	0.43±0.09	0.72±0.23	7.56±1.83
T		0.218	0.398	5.441
P		>0.05	>0.05	<0.05

### Feeding outcomes

The treatment group had significantly higher milk amount and calorie intake than those of the control group (P<0.05), and neonates in the treatment group who reached calorie intake of 50/80/100 kcal/kg/d were significantly younger (P<0.05). Meanwhile, the treatment group suffered from significantly less feeding intolerance symptoms than the control group did (P<0.05) (Table-III).

**Table-III:** Feeding outcomes.

Index	Treatment group (n=95)	Control group (n=95)	t or χ^2^	P
Milk amount	95.63±6.98	81.83±7.01	5.982	<0.05
Calorie	76.29±5.44	62.87±10.23	6.001	<0.05
Ages in days on which 50 kcal/kg/d calories were reached	4.41±0.72	6.65±0.89	5.008	<0.05
Ages in days on which 80 kcal/kg/d calories were reached	7.43±1.26	10.98±1.63	6.918	<0.05
Ages in days on which 100 kcal/kg/d calories were reached	9.86±2.38	13.98±2.74	8.745	<0.05
Feeding intolerance	12 (12.6%)	34 (35.8%)	5.093	<0.05

### Serum gastrointestinal hormone levels

After 14 days of feeding, the serum MOT and GAS levels of both groups rose, with significant intra-group and inter-group differences. Such levels of the treatment group significantly exceeded those of the control group (P<0.05) (Table-IV).

**Table-IV:** Serum gastrointestinal hormone levels (pg/ml, x±s).

Group	Case No. (n)	MOT		GAS	
		On 1st day of feeding	After 14 days of feeding	On 1st day of feeding	After 14 days of feeding
Treatment group	95	635.33±89.34	933.76±83.22	624.87±54.93	856.93±45.88
Control group	95	633.97±78.22	836.98±87.33	620.65±60.48	734.56±60.42
t		0.145	3.092	0.089	3.221
P		>0.05	<0.05	>0.05	<0.05

### Serum vitamin D levels

The serum vitamin D levels of both groups increased after 14 days of feeding, and the level of the treatment group was significantly higher (P<0.05) (Table-V).

**Table-V:** Serum vitamin D levels (nmol/L, x±s).

Group	Case No. (n)	On 1st day of feeding	After 14 days of feeding
Treatment group	95	16.37±5.29	20.78±5.44
Control group	95	16.44±6.09	18.76±4.22
t		0.137	3.744
P		>0.05	<0.05

## DISCUSSION

Neonates are enjoying increasing survival rate due to development of diagnosis, treatment and nursing technologies, and particular attention has also been paid to their quality of life. The nutritional status of neonates affects their growth and development, and nutrient deficiencies exert adverse effects on the recovery of diseases in them, especially in premature infants. Thus, their feeding has been spotlighted.^11^ The brain development of neonates is regulated by proteins, energy intake, long-chain polyunsaturated fatty acids, vitamins and minerals. Malnutrition during the neonatal period, which may affect the cognitive function when they grow up, can be circumvented by supplementing many types of micronutrients.^12^,^13^ Since most neonates in this study were premature infants, they had low birth weights and limitedly digested carbohydrates. Meanwhile, low weight generally meant small stomach capacity and less food intake, which affected the intake of nutrients and calories, leading to feeding intolerance by easily inducing reflux, choking and aspiration pneumonia.

Early micro-feeding can promote the gastrointestinal development and maturation of neonates, premature infants in particular, allowing more milk amount and calorie intake as well as meeting their requirements.^14^ In this study, formula milk was prepared according to the special characteristics of the digestive system of the premature infants and their requirements for energy and nutrients, which provided sufficient nutrition and benefited the development and maturation of their gastrointestinal function.^15^ It is well-documented that glutamine can improve metabolism and immune function, affect intestinal function and enhance stress response.^16^ The weight of the treatment group herein increased significantly faster than that of the control group did (P<0.05), but their head circumferences and heights grew at similar rates. The treatment group had significantly higher milk amount and calorie intake than those of the control group (P<0.05), and neonates in the treatment group who reached calorie intake of 50/80/100 kcal/kg/d were significantly younger (P<0.05). In the meantime, the treatment group suffered from significantly less feeding intolerance symptoms than the control group did (P<0.05). Hence, adding glutamine in early enteral nutritional intervention was conducive to digestion and absorption of nutrients together with synthesis of glycogen, proteins and lipids, thereby accelerating weight gain.

Gastrointestinal hormones, serum MOT and GAS, are involved in the development and maturation of the digestive tract of neonates, particularly in the improvement of gastrointestinal structure and function.^17^ Mainly secreted by M cells in the small intestine, MOT is primarily responsible for promoting gastrointestinal motility. GAS, which is mainly secreted by G cells in the gastrointestinal tract, stimulates secretion of gastric acid, pepsin and trypsin as well as provides nutrients for basal cells in the gastrointestinal mucosa. As the main energy source for intestinal mucosal cells and all the rapidly growing cells, glutamine is beneficial to secretion of gastrointestinal hormones in the disease state.^18^ After 14 days of feeding, the serum MOT and GAS levels of both groups rose, with significant intra-group and inter-group differences. Such levels of the treatment group significantly surpassed those of the control group (P<0.05).

Besides being self-synthesized by human body, vitamin D can also be acquired from food or pharmaceutical preparations, absorbed by mucosal cells in the small intestine and transformed into the active form through secondary hydroxylation to play biological roles.^19^ Vitamin D, when abundant after intake or sunshine-induced synthesis, can be stored in fatty tissues, skeletal muscle and the liver for future use.^20^ In this study, the serum vitamin D levels of both groups rose after 14 days of feeding, and the level of the treatment group was also significantly higher (P<0.05), suggesting that adding glutamine in early micro-feeding enhanced the activity of gastrointestinal enzymes and increased gastrointestinal blood flow. As a result, vitamin D was better absorbed and stored owing to increased feeding tolerance.

## CONCLUSION

Providing enteral nutrition support for neonates pave the way for their growth and development, supplementing which with glutamine can promote the absorption of routine nutrients and vitamin D, apparently regulate gastrointestinal hormones, and facilitate weight gain.
